# SATB2, coordinated with CUX1, regulates IL‐1β‐induced senescence‐like phenotype in endothelial cells by fine‐tuning the atherosclerosis‐associated p16^INK4a^ expression

**DOI:** 10.1111/acel.13765

**Published:** 2023-01-12

**Authors:** Ting Wu, Yuwei Wu, Danli Jiang, Wei Sun, Meijuan Zou, Sathish Babu Vasamsetti, Partha Dutta, Steven A. Leers, Wu Di, Gang Li

**Affiliations:** ^1^ Department of Cardiovascular Medicine, Xiangya Hospital Central South University Changsha Hunan China; ^2^ Aging Institute University of Pittsburgh Pittsburgh Pennsylvania USA; ^3^ Department of Medicine, Xiangya School of Medicine Central South University Changsha Hunan China; ^4^ Center for Pulmonary Vascular Biology and Medicine, Pittsburgh Heart, Lung, Blood, and Vascular Medicine Institute University of Pittsburgh School of Medicine and University of Pittsburgh Medical Center Pennsylvania Pittsburgh USA; ^5^ UPMC Vascular Laboratories University of Pittsburgh Medical Center Pittsburgh Pennsylvania USA; ^6^ Department of Periodontology University of North Carolina at Chapel Hill Chapel Hill North Carolina USA; ^7^ Department of Medicine, Division of Cardiology University of Pittsburgh Medical Center Pittsburgh Pennsylvania USA

**Keywords:** aging, aging‐related diseases, atherosclerosis, cellular senescence, endothelial cells, post‐GWAS functional analysis, senescence‐associated secretory phenotype (SASP), single nucleotide polymorphism (SNP)

## Abstract

Genome‐wide association studies (GWAS) have validated a strong association of atherosclerosis with the *CDKN2A/B* locus, a locus harboring three tumor suppressor genes: *p14* ^*ARF*^, *p15* ^*INK4b*^, and *p16* ^*INK4a*^. Post‐GWAS functional analysis reveals that CUX is a transcriptional activator of p16^INK4a^ via its specific binding to a functional SNP (fSNP) rs1537371 on the atherosclerosis‐associated *CDKN2A/B* locus, regulating endothelial senescence. In this work, we characterize SATB2, another transcription factor that specifically binds to rs1537371. We demonstrate that even though both CUX1 and SATB2 are the homeodomain transcription factors, unlike CUX1, SATB2 is a transcriptional suppressor of p16^INK4a^ and overexpression of SATB2 competes with CUX1 for its binding to rs1537371, which inhibits p16^INK4a^ and p16^INK4a^‐dependent cellular senescence in human endothelial cells (ECs). Surprisingly, we discovered that SATB2 expression is transcriptionally repressed by CUX1. Therefore, upregulation of CUX1 inhibits SATB2 expression, which enhances the binding of CUX1 to rs1537371 and subsequently fine‐tunes p16^INK4a^ expression. Remarkably, we also demonstrate that IL‐1β, a senescence‐associated secretory phenotype (SASP) gene itself and a biomarker for atherosclerosis, induces cellular senescence also by upregulating CUX1 and/or downregulating SATB2 in human ECs. A model is proposed to reconcile our findings showing how both primary and secondary senescence are activated via the atherosclerosis‐associated p16^INK4a^ expression.

## Graphical abstract

Previously, we identified CUX1 as an activator of p16^INK4a^‐dependent cellular senescence in response to telomere shortening, DNA damage, and oxidative stress via its specific binding to an atherosclerosis‐associated fSNP rs1537371 on the *CDKN2A/B* locus. In this manuscript, we demonstrate that SATB2 is a suppressor of p16^INK4a^‐dependent cellular senescence by binding to the same SNP. Further analysis reveals that CUX1 is a transcriptional repressor of SATB2, regulating cellular senescence by fine‐tuning p16^INK4a^ expression in response to IL‐1ß.

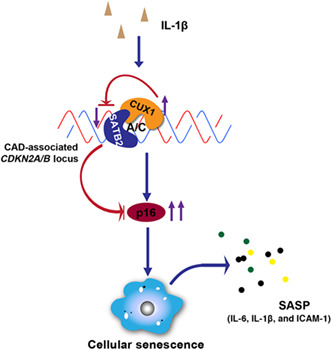

## 1 INTRODUCTION

Cellular senescence is defined as an irreversible cell cycle arrest. It is a phenomenon characterized by the cessation of cell division often accompanied by an enlarged and flattened cellular morphology. In association with the arrest, senescent cells also secrete multiple proinflammatory molecules, growth factors, and proteases such as the interleukins IL‐6 and IL‐1β, intercellular adhesion molecule 1 (ICAM‐1), vascular cell adhesion molecule1 (VCAM‐1), granulocyte macrophage colony‐stimulating factor (GM‐CSF), vascular endothelial growth factor (VEGF), and matrix metalloproteinase 8 (MMP8), collectively known as the senescence‐associated secretory phenotype (SASP). Based on the initiating trigger, cellular senescence can be classified as replicative or stress‐induced senescence (Armanios & Blackburn, 2012; Hayflick & Moorhead, 1961). Both replicative and stress‐induced senescence are mediated through p53/p21 and/or p16^INK4a^/RB (retinoblastoma protein) pathways; however, preference for one pathway over the other depends on cell type, species and the stimuli (Gorgoulis et al., 2019). Also, both replicative and stress‐induced senescence are now recognized as primary senescence in contrast to secondary senescence that is induced by primary senescent cells either via cell‐to‐cell direct contact (juxtacrine), or through the secretion of SASP (paracrine) (Admasu et al., 2021).

Increasing evidence demonstrates that the accumulation of senescent cells with age is a main contributor to aging and age‐related diseases (Campisi & Robert, 2014; Childs et al., 2015). Induction of p16^INK4a^ has been reported to lead to cellular senescence in a variety of cells and tissues (Kanavaros et al., 2001; Nielsen et al., 1999; Zindy et al., 1997). Depletion of p16^INK4a^‐positive cells in either a normal mouse model or a mouse model with accelerated aging using genetic manipulation or synolytic reagents eliminate senescent cells in different organs and tissues, which delays age‐related diseases and extends healthy life span (Baker et al., 2011, 2016; Dang et al., 2020; Hickson et al., 2019). These findings suggest that p16^INK4a^ plays a pivotal role in aging and age‐related diseases. In addition, p16^INK4a^ is also one of the markers for cellular senescence and other senescent markers include SA‐β‐gal (senescence‐associated β‐galactosidase), γ‐H2AX (H2A histone family member X), telomeric length, the expression of SASP genes and cell cycle arrest.

*p16* ^*INK4a*^ is one of the tumor suppressor genes located in the *CDKN2A/B* locus on chromosome 9p21. The other genes that are located at this locus are tumor suppressor genes *p14* ^*ARF*^, *p15* ^*INK4b*^ as well as a long noncoding RNA called ANRIL (antisense noncoding RNA in the inhibitor of CDK4 (INK4) locus). Consistent with the role of p16^INK4a^ in aging and age‐related pathologies, GWAS have demonstrated a strong association of the *CDKN2A/B* locus with multiple pathologies such as cardiovascular diseases including coronary artery disease (CAD), myocardial infarction, aneurysms, peripheral artery disease, and heart failure, in addition to other diseases such as glaucoma, type 2 diabetes, and various forms of cancer (Hannou et al., 2015; Kong et al., 2016). Of note, all these disorders are recognized as age‐related diseases in that their incidence markedly increases as a function of age. The same *CDKN2A/B* locus was also associated with frailty and overall human life span (Fortney et al., 2015; Giuliani et al., 2018). However, very few studies have tried to mechanistically investigate how disease‐associated causative fSNPs in the *CDKN2A/B* locus regulate the expression of the *CDKN2A/B* genes including *p16* ^*INK4a*^ in the context of cellular senescence.

IL‐1β is an inflammatory molecule that belongs to the interleukin cytokine 1 family. It is an important mediator of the inflammatory response and is involved in a variety of cellular processes, including cell proliferation, differentiation, and apoptosis (Libby, 2017). *IL‐1β* is also a SASP gene that is activated in senescent cells (Coppe et al., 2008). Ample evidence has been reported to show the important role that IL‐1β plays in atherosclerosis. For example, high levels of plasma IL‐1β were detected in patients with atherosclerosis, which are positively correlated with the severity of the disease (Qamar & Rader, 2012) and inhibition of IL‐1β‐induced signal transduction alleviates atherosclerosis (Libby, 2017; Ridker et al., 2017). Moreover. IL‐1β has different effects on different types of cells involved in atherosclerosis (Libby, 2021). In endothelial cells, IL‐1β was reported to induce adhesion molecules such as VCAM‐1 and ICAM‐1. These molecules could be responsible for recruiting inflammatory monocytes, which promotes their invasion into local intima, a step that occurs at the initiation of atherosclerosis (Bevilacqua et al., 1985). IL‐1β also induces cytokines such as IL‐6 regulating different immune responses (Loppnow & Libby, 1990). Of note, all these molecules are now recognized as SASP genes, which suggests that IL‐1β is an inducer of cellular senescence. Consistent with this note, IL‐1β was demonstrated as an inducer of cellular senescence in cancer cells (Hubackova et al., 2012), astrocytes (Shang et al., 2020), vascular smooth muscle cells (VSMCs) (Han et al., 2020; Shang et al., 2020), and chondrocytes (Chai et al., 2020; Huang et al., 2018; Philipot et al., 2014). However, little is known about the signaling pathway that regulates IL‐1β‐induced senescence.

Previously, using Reel‐seq and FREP‐MS, two novel techniques developed in our laboratory (Li et al., 2018; Zhao et al., 2020), we identified six proteins including CUX1, SATB1, SATB2, HOXA10, NFIC, and MYH9 specifically binding to a fSNP rs1537371 on the *CDKN2A/B* locus (Jiang et al., 2022). Among these proteins, we demonstrated that CUX1, a homeodomain transcription factor, activates both replicative and stress‐induced senescence by upregulating p16^INK4a^ expression (Jiang et al., 2022). In this report, we show that SATB2, another homeodomain transcription factor, suppresses cellular senescence in human arterial ECs by downregulating p16^INK4a^ expression via its competitive binding with CUX1 to rs1537371. At the same time, we also demonstrate that CUX1 is a transcriptional repressor of SATB2 since upregulation of CUX1 decreases SATB2 expression. Most important, we discover that IL‐1β can activate a senescence‐like phenotype by fine‐tuning p16^INK4a^ expression. This is performed by upregulating CUX1 and downregulating SATB2 in human ECs.

## 2 RESULTS

### 2.1 Demonstration of allele‐imbalanced binding of SATB2 to the atherosclerosis‐associated fSNP rs1537371 on the *CDKN2A/B* locus in human arterial ECs

**FIGURE 1:**
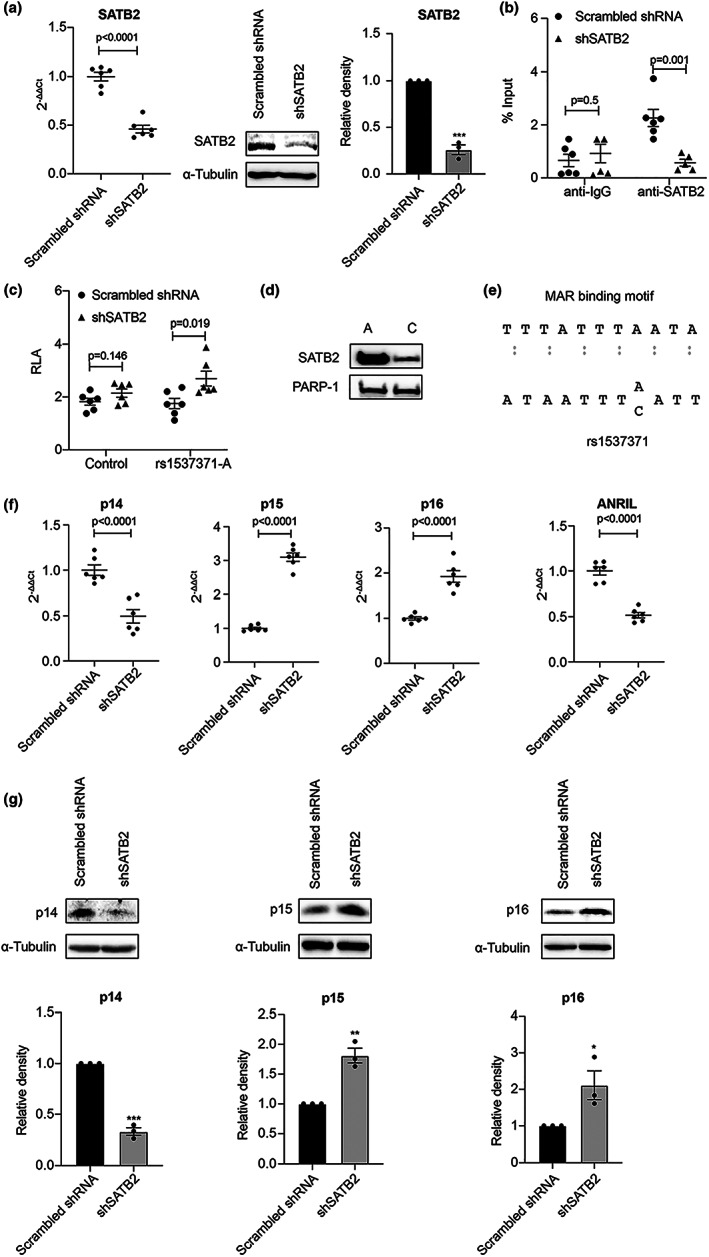
SATB2 regulates the expression of *CDKN2A/B* genes via its specific binding to the fSNP rs1537371. (a) qPCR and Western blot demonstrating downregulation of *SATB2* by shRNA in primary human arterial ECs. Relative density of SATB2 in the Western blot was shown. Data for Western blots represent three biologically independent experiments (*n* = 3). Data for qPCR analysis represent a combination of three biologically independent samples (*n* = 3), each performed in duplicate. (b) ChIP assay demonstrating a decreased binding of SATB2 to rs1537371 in the SATB2 shRNA knockdown ECs. Data for ChIP assay represent three biologically independent experiments (*n* = 3). Rabbit anti‐IgG antibody was used as a control. (c) SATB2‐dependent luciferase reporter assay showing an upregulation of luciferase activities under the conditions of shRNA *SATB2* knockdown in 293 T cells. RLA: relative luciferase activity; rs1537371‐A and Con: luciferase reporter construct pGL3 (Promoter vector, Promega) containing the fSNP rs1537371 with the risk allele A or an irrelevant SNP as a negative control. Data for this assay represent six biologically independent samples (*n* = 6). (d) AIDP‐Wb showing that SATB2 binds to the fSNP rs1537371 in an allele‐imbalanced manner with the risk allele A binding more SATB2 than the non‐risk allele C. PARP‐1, a DNA end‐binding protein, was used as an internal loading control. Data for AIDP‐Wb represent three biologically independent experiments (*n* = 3). (e) Sequence analysis showing the sequence similarity between the MAR‐binding motif and the fSNP rs1537371 surrounding sequence. (f) qPCR showing a downregulation of *p14* ^*ARF*^ and *ANRIL*, and an upregulation of *p15* ^*INK4b*^ and *p16* ^*INK4a*^ in the *SATB2* shRNA knockdown human ECs. Data for qPCR analysis represent a combination of three biologically independent samples (*n* = 3), each performed in duplicate. (g) Western blot analysis showing a downregulation of *p14* ^*ARF*^, and an upregulation of *p15* ^*INK4b*^, and *p16* ^*INK4a*^ in the *SATB2* shRNA knockdown human ECs. Relative density of p14^ARF^, p15^INK4b^, and p16^INK4a^ in the Western blots was shown. Data for Western blot analysis represent three biologically independent experiments (*n* = 3). sh: shRNA. α‐Tubulin is used as a loading control. *p‐value < 0.05; **p‐value < 0.01; ***p‐value < 0.001.

Previously, we identified that homeodomain transcription factor CUX1, as one of the six proteins in a complex, activates p16^INK4a^‐dependent cellular senescence via its specific binding to the atherosclerosis‐associated fSNP rs1537371 on the *CDKN2A/B* locus in human arterial ECs (Jiang et al., 2022). Another one of these six proteins in the complex is SATB2, also known as a homeodomain transcription factor (Britanova et al., 2005). To determine whether SATB2 is also a regulator of cellular senescence, we first demonstrated the specific binding of SATB2 to the fSNP rs1537371 using chromatin immunoprecipitation (ChIP) assay in the SATB2 shRNA knockdown ECs. Knockdown of SATB2 was first confirmed by both qPCR and Western blot as shown in Figure 1a. As a result, a significant decrease in the DNA fragment containing the fSNP rs1537371 was detected in the *SATB2* shRNA knockdown ECs compared to the scrambled shRNA control cells (*p*‐value = 0.001) (Figure 1b, right), indicating that there is a specific endogenous binding of SATB2 to the fSNP rs1537371. As a negative control, we performed the same ChIP assay using an isotype control antibody (an anti‐IgG antibody), and no obvious difference was observed between the SATB2 shRNA knockdown ECs and the control ECs treated with scrambled shRNA (*p*‐value = 0.5) (Figure 1b, left). Next, we performed a luciferase reporter assay using a reporter construct that contains the risk allele A from the fSNP rs1537371 also in the SATB2 shRNA knockdown cells. This reporter construct was previously used to validate the fSNP rs1537371 by detecting the allele‐imbalanced luciferase activity that shows the risk allele A having more luciferase activity than the nonrisk allele C (Jiang et al., 2022). Our data, as shown in Figure 1c (right), indicate that downregulation of SATB2 resulted in a significantly increased luciferase activity (*p*‐value = 0.019) by comparing the SATB2 shRNA knockdown cells with the scrambled shRNA control cells. While this result demonstrates the specific binding of SATB2 to the fSNP rs1537371, it also suggests that SATB2 acts as a suppressor as knockdown of SATB2 increases the luciferase reporter activity. Also, as a negative control, we performed the same luciferase reporter assay with a reporter construct containing an SNP sequence from an irrelevant SNP and as expected, we did not observe any significant difference in luciferase activity between the SATB2 shRNA knockdown cells and the scrambled shRNA control cells (*p*‐value = 0.146) (Figure 1c, left).

Even though both the ChIP assay and the luciferase reporter assay detected the specific binding of SATB2 to the DNA fragment containing the fSNP rs1537371, this does not necessarily indicate a specific binding of SATB2 to the fSNP rs1537371 itself unless an allele‐imbalanced binding of SATB2 to the fSNP rs1537371 can be demonstrated. To prove this, we applied allele‐imbalanced DNA pulldown‐Western blot (AIDP‐Wb), a novel method that was recently developed in our laboratory to detect the interaction between a known protein and a confirmed fSNP (Zhao et al., 2020). Using this method, a differential binding of SATB2 with the risk allele A binding more SATB2 than the nonrisk allele C was observed (Figure 1d). In addition, the specific binding of SATB2 to the fSNP rs1537371 was further demonstrated by the sequence similarity between the surrounding sequence ATAATTT[A/C]ATT from the fSNP rs1537371 and the MAR (matrix attachment region)‐binding motif TTTATTTAATA as SATB2 is a MAR binding protein (Figure 1e) (Alvarez et al., 2000). Together, these data demonstrate that SATB2 is a protein that specifically binds to the fSNP rs1537371 in an allele‐imbalanced manner.

### 2.2 SATB2 regulates the expression of p14^ARF^ , p15^INK4b^ , p16^INK4a^, and ANRIL in human arterial ECs

Given that the atherosclerosis‐associated fSNP rs1537371 is located in the *CDKN2A/B* locus that harbors *p14* ^*ARF*^ *, p15* ^*INK4b*^ *, p16* ^*INK4a*^, and ANRIL (Hannou et al., 2015), we investigated the expression of these four genes in the same *SATB2* shRNA knockdown human ECs as we showed in Figure 1a. Compared with the scrambled shRNA control ECs, knockdown of *SATB2* significantly decreased the expression of both *p14* ^*ARF*^ and ANRIL, whereas the expression of *p15* ^*INK4b*^ and *p16* ^*INK4a*^ was significantly upregulated as detected at the mRNA level (Figure 1f). The same pattern expression of p14^ARF^, p15^INK4b^, and p16^INK4a^ proteins in the *SATB2* knockdown human ECs was also detected by Western blots (Figure 1g). To further confirm that SATB2 transcriptionally regulates *p14* ^*ARF*^ *, p15* ^*INK4b*^ *, p16* ^*INK4a*^, and ANRIL, we also transiently transfected an SATB2 siRNA into human ECs. This siRNA targets a sequence of *SATB2* that is different from that used in the *SATB2* shRNA knockdown. A similar result showing a downregulation of both *p14* ^*ARF*^ and ANRIL as well as an upregulation of both *p15* ^*INK4b*^ and *p16* ^*INK4a*^ was detected by qPCR (Figure S1a). Thus, both *SATB2* shRNA and siRNA knockdowns demonstrate that SATB2 is a transcriptional regulator that controls the expression of *p14* ^*ARF*^ *, p15* ^*INK4b*^ *, p16* ^*INK4a*^, and ANRIL in human ECs. Since p14^ARF^, p15^INK4b^, and p16^INK4a^ are cell cycle inhibitors regulating cell proliferation, we also performed cell proliferation assay and cell cycle analysis in the *SATB2* shRNA knockdown human ECs and observed a defect in cell proliferation (Figure S1b) and a decrease in cell numbers in the S/G_2_/M phase in these ECs (Figure S1c).

These data, together with the data presented in Figure 1a–e, demonstrate that SATB2 is a transcriptional regulator modulating the expression of *p14* ^*ARF*^ *, p15* ^*INK4b*^ *, p16* ^*INK4a*^, and ANRIL via binding to the fSNP rs1537371 at the *CDKN2A/B* locus.

### 2.3 SATB2 suppresses cellular senescence in human ECs by downregulating p16^INK4a^ expression

**FIGURE 2:**
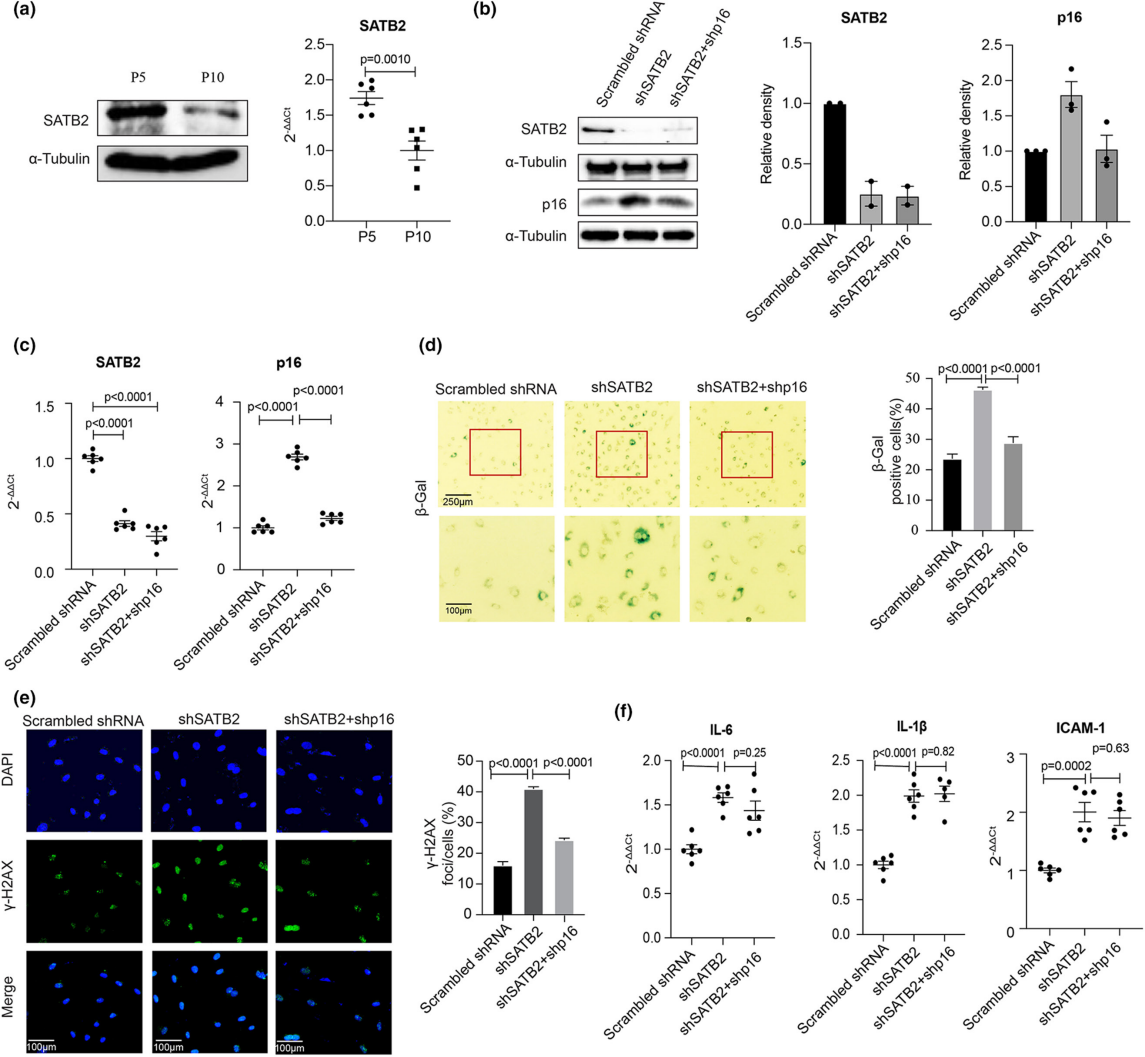
Functional analysis demonstrating that downregulation of SATB2 induces cellular senescence by activating p16^INK4a^ expression. (a) Western blot (left) and qPCR (right) demonstrating that SATB2 expression is downregulated in passage 10 ECs versus passage 5 ECs. (b) Western blot and (c) qPCR analysis showing a significant upregulation of p16^INK4a^ in the *SATB2* shRNA knockdown ECs (left and middle lane), as well as the reversed expression of p16^INK4a^ in the *SATB2 and p16* ^*INK4a*^ double knockdown human ECs (middle and right lane). Relative density of SATB2 and p16^INK4a^ in the Western blots was shown. Data for SATB2 Western blot analysis represent two biologically independent experiments (*n* = 2). Data for p16^INK4a^ Western blot analysis represent three biologically independent experiments (*n* = 3). Data for qPCR analysis represent a combination of three biologically independent samples (*n* = 3), each performed in duplicate. (d) SA‐β‐gal and (e) γ‐H2AX staining showing an increased cellular senescence in the *SATB2* shRNA knockdown ECs (left and middle panel) and a recovery of cellular senescence in the *SATB2* and *p16* ^*INK4a*^ double knockdown human ECs (middle and right panel). DAPI (blue) was applied to stain nuclei. Quantitative plots for both β‐gal‐positive cells (%) following SA‐β‐gal staining and γ‐H2AX foci/cells (%) after γ‐H2AX staining are shown on the right side of the panel. Data for SA‐β‐gal and γ‐H2AX staining represent three biologically independent experiments (*n* = 3). (f) qPCR analysis showing the upregulation of the SASP genes *IL‐6*, *IL‐1β* and *ICAM‐1* in the *SATB2* shRNA knockdown human ECs (left and middle lane). However, downregulation of p16^INK4a^ by shRNA knockdown did not reduce the increased expression of *IL‐6*, *IL‐1β* and *ICAM‐1* in the *SATB2* shRNA knockdown ECs (middle and right lane). Data for qPCR analysis represent a combination of three biologically independent samples (*n* = 3), each performed in duplicate. Sh: shRNA.

Among the four genes located at the *CDKN2A/B* locus, *p16* ^*INK4a*^ has been implicated in cellular senescence (Baker et al., 2011, 2016). Upregulation of p16^INK4a^ (Figure 1f,g) in the *SATB2* knockdown ECs suggests that SATB2 could be a suppressor of cellular senescence via downregulating p16^INK4a^ expression. To test this hypothesis, we first investigated the possible role of SATB2 in replicative senescence by measuring the expression of SATB2 in passage 5 versus passage 10 ECs. As the results can be seen in Figure 2a, a significant decrease in SATB2 expression in the passage 10 ECs was detected, suggesting that SATB2 is a suppressor of cellular senescence. This result is consistent with our previous findings that CUX1 is an activator of cellular senescence and its expression is significantly increased in p10 ECs compared to p5 ECs (Jiang et al., 2022). We next confirmed the upregulated expression of p16^INK4a^ in the *SATB2* shRNA knockdown human ECs at passage 8 by both Western blot (Figure 2b, left and middle lane) and qPCR analysis (Figure 2c, left and middle lane). In comparison with the scrambled shRNA human ECs, the *SATB2* shRNA knockdown human ECs showed a significant increase in senescence markers including both the SA‐β‐gal and the γ‐H2AX stainings (Figure 2d,e, left and middle panel), as well as the expression of the SASP genes *IL‐6*, *IL‐1β*, and *ICAM‐1* (Figure 2f, left and middle lane) two days after shRNA lentiviral infection. We also performed the same SA‐β‐gal staining five days after the shRNA lentiviral infection and the same results were observed (Figure S2). These data indicate that SATB2 is a suppressor of cellular senescence in human ECs and downregulation of SATB2 results in an induction of cellular senescence. To demonstrate that SATB2 suppresses cellular senescence via downregulating the p16^INK4a^ expression, we performed an shRNA knockdown of *p16* ^*INK4a*^ in the *SATB2* shRNA knockdown ECs as shown in Figure 2b,c (middle and right lane). Compared with the *SATB2* shRNA knockdown ECs, these *SATB2* and *p16* ^*INK4a*^ double knockdown ECs showed a restoration of cellular senescence to a level like that of the scrambled shRNA knockdown ECs as evidenced by reduced SA‐β‐gal and γ‐H2AX staining (Figure 2d,e, middle and right lane). These data demonstrate that p16^INK4a^ is a transcriptional target of SATB2, and that SATB2 suppresses cellular senescence by downregulating the p16^INK4a^ expression. However, the expression of the SASP genes *IL‐6*, *IL‐1β* and *ICAM‐1* remained unchanged (Figure 2f, middle and right lane) in the *SATB2* and *p16* ^*INK4a*^ double knockdown human ECs. This result is intriguing but consistent with previous publications demonstrating that p16^INK4a^ is not an SASP factor responsible for SASP gene expression (Coppe et al., 2011).

To further demonstrate that SATB2 is a suppressor of cellular senescence by inactivating p16^INK4a^ expression, we first perofmed a functional complementation assay by ectopically overexpressing *p16^INK4a^* in the *SATB2* overexpressed human ECs at passage 10. (Figure S3a). Consistently, we observed a restoration of cellular senescence as detected by SA‐β‐gal and γ‐H2AX staining as well as the expression of the SASP genes *IL‐6*, *IL‐1β*, and *ICAM‐1* (Figure S3b–d). Next, we performed an immunocytochemical staining on the plaques of pateints with carotid artery disease using antibodies specifically against both SATB2 and p16^INK4a^. As we know, atherosclerosis is considered as an age‐related disease and cellular senescence has been detected in the human arterial ECs of patients with atherosclerosis (Katsuumi et al., 2018; Minamino et al., 2002). Again, consistently, we observed a reduced SATB2 and an induced p16^INK4a^ staining in the plaque zones when comparing them to the normal‐appearing zones even though the overall expression level of SATB2 is low in normal‐appearing zones (*n* = 8) (Figure S4).

In addition, like p16^INK4a^, p15^INK4b^ is also upregulated in the *SATB2* knockdown human ECs as we showed; therefore, we tested the possibility that SATB2 may also suppress cellular senescence via inactivating p15^INK4b^. However, our data suggest that p15^INK4b^ is not responsible for cellular senescence at least in the *SATB2* shRNA knockdown human ECs (Figure S5). We also tested whether ANRIL is involved in regulating cellular senescence in human ECs as recent publications suggested (Muniz et al., 2021; Tan et al., 2019). However, once more, we did not observe any obvious change in neither SA‐β‐gal nor γ‐H2AX staining in the ANRIL siRNA knockdown human ECs (Figure S6).

### 2.4 SATB2, repressed by CUX1, competes with CUX1 for binding to the fSNP rs1537371 in human ECs

**FIGURE 3:**
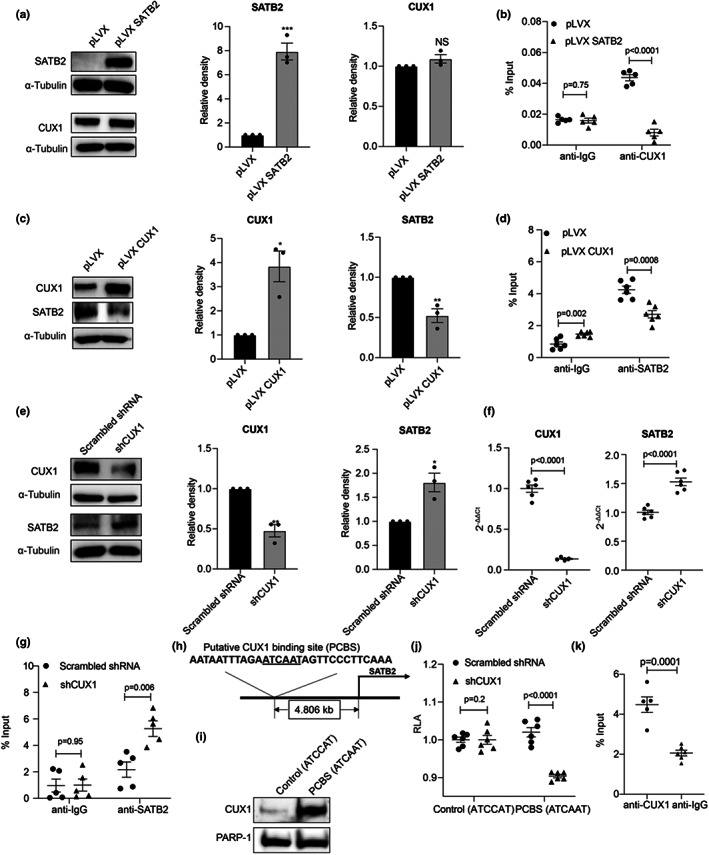
SATB2, repressed by CUX1, competes with CUX1 for its binding to the fSNP rs1537371 in human ECs. (a) Western blot analysis showing no obvious change of CUX1 expression in the SATB2‐overexpressed human ECs. Data for the Western blot analysis represent three biologically independent experiments (*n* = 3). (b) ChIP assay using an anti‐CUX1 antibody demonstrating that the binding of CUX1 to the fSNP rs1537371 is significantly reduced (*p*‐value <0.0001) in the SATB2‐overexpressed human ECs (right). As a control, anti‐IgG isotype antibody was used, and no obvious change was observed (*p*‐value = 0.75) (left). Data for ChIP assay represent three biologically independent samples (*n* = 3), each performed in duplicate. (c) Western blot analysis showing a downregulation of SATB2 expression in the CUX1‐overexpressed human ECs. Data for the Western blot analysis represent three biologically independent experiments (*n* = 3). (d) ChIP assay using an anti‐SATB2 antibody demonstrating that the binding of SATB2 to the fSNP rs1537371 is significantly reduced (*p*‐value = 0.0008) in the CUX1 overexpressed human ECs (right). As a control, anti‐IgG isotype antibody was used (left). Data for ChIP assay represent three biologically independent samples (*n* = 3), each performed in duplicate. (e) Western blots and (f) qPCR showing an upregulation of SATB2 expression in the CUX1 shRNA knockdown human ECs. Data for the Western blot analysis represent three biologically independent experiments (*n* = 3). Data for qPCR analysis represent a combination of three biologically independent samples (*n* = 3), each performed in duplicate. (g) ChIP assay using an anti‐SATB2 antibody demonstrating that the binding of SATB2 to the fSNP rs1537371 is significantly increased (*p*‐value = 0.006) in the CUX1 shRNA knockdown human ECs (right). As a control, anti‐IgG isotype antibody was used, and no change was observed (*p‐*value =0.95) (left). Data for ChIP analysis represent three biologically independent samples (*n* = 3), each performed in duplicate. (h) Diagram showing the sequence of a putative CUX1 binding site (PCBS) and its relative location (4.8 kb upstream of the SATB2 transcription start site) within the *SATB2* promoter region. Underlined nucleotides represent the CUX1 core binding motif ATCAAT. (i) DNA pulldown Western blot analysis demonstrating a specific binding of CUX1 to the 30‐bp PCBS containing the CUX1 core binding motif ATCAAT. As a control, PCBS containing a mutated CUX1 core binding motif ATCCAT was used. PARP‐1 was used as an internal loading control. (j) CUX1‐dependent luciferase reporter assay showing a downregulation of luciferase activity in the *CUX1* shRNA knockdown 293 T cells. RLA: relative luciferase activity; PCBS (ATCAAT): luciferase reporter construct pGL3 (Promoter vector, Promega) containing PCBS with the CUX1 core binding motif ATCAAT. Control (ATCCAT): a control luciferase reporter construct pGL3 containing a mutated CUX1 core binding motif ATCCAT that was used as a negative control. Data for this assay represent six biologically independent samples (*n* = 6). (k) ChIP assay in ECs using an anti‐CUX1 antibody showing an increased binding of CUX1 to the putative CUX1 binding site (PCBS) on the *SATB2* promoter. For negative control anti‐IgG antibody was used. pLVX: overexpression vector control; pLVX SATB2 and pLVX CUX1: overexpression of SATB2 and CUX1; and sh: shRNA. Relative density of CUX1 and SATB2 in all the Western blots was shown. *p‐value < 0.05; **p‐value < 0.01; ***p‐value < 0.001. For control, an anti‐IgG isotype antibody was used.

We previously showed that SATB2 and CUX1 were pulled out in the same protein complex using a 31 bp DNA fragment containing the fSNP rs1537371 as a “bait.” (Jiang et al., 2022). Like SATB2, CUX1 also binds to rs1537371 with the risk allele binding more than the non‐risk allele; however, CUX1 is an activator of p16^INK4a^ and p16^INK4a^‐dependent endothelial senescence (Jiang et al., 2022). Based on these data, we hypothesize that SATB2 might compete with CUX1 for its binding to the fSNP rs1537371 and then suppressing p16^INK4a^ and p16^INK4a^‐dependent endothelial senescence. To test this hypothesis, we first performed a ChIP assay using an anti‐CUX1 antibody on the DNA fragment containing the fSNP rs1537371 in human ECs, in which SATB2 is ectopically overexpressed using pLVX‐SATB2 vector. Western blot analysis confirmed the overexpression of SATB2 in human ECs, whereas there is no change of CUX1 expression (Figure 3a). As shown in Figure 3b (right), a decreased binding of CUX1 to the rs1537371‐containing DNA fragment was detected in the SATB2 overexpressed ECs compared to the control ECs transfected with pLVX‐puro vector (Clontech, Cat#: 632164) (*p*‐value = 0.0001). As a negative control, the same ChIP assay was performed using an anti‐IgG isotype antibody, and no obvious difference was observed (*p*‐value = 0.75) (Figure 3b, left). These data clearly demonstrate that SATB2 competes with CUX1 for its binding to the fSNP rs1537371 and overexpression of SATB2 decreases the binding of CUX1 to the fSNP rs1537371 without changing the level of CUX1 expression in human ECs.

To further demonstrate that SATB2 competes with CUX1 for its binding to the fSNP rs1537371, we performed the same ChIP assay; however, we used an anti‐SATB2 antibody in the CUX1 overexpressed human ECs. To our surprise, in the CUX1 overexpressed human ECs, a decreased expression of SABT2 was observed (Figure 3c), which suggests that CUX1 is a suppressor of SATB2. Consistent with this expression profile, a significant reduction in the binding of SATB2 to the fSNP rs1537371‐containing DNA fragment was detected in the CUX1‐overexpressed ECs compared to the control ECs transfected with pLVX‐puro vector (Figure 3d, right). To further confirm that CUX1 is a negative regulator of SATB2, we performed a CUX1 shRNA knockdown in human ECs. Not surprising, a significant upregulation of SATB2 was detected at both the protein and mRNA levels as shown in Figure 3e,f. Consistent with this expression profile and, also as expected, we identified a significant increase in the binding of SATB2 to the DNA fragment containing fSNP rs1537371 in the ChIP assay using the anti‐SATB2 antibody (Figure 3g).

To further demonstrate that CUX1 is a transcription factor suppressing SATB2 expression, we searched the genomic sequence on the promotor region of *SATB2* and identified a CUX1 core binding motif ATCAAT (Vadnais et al., 2013) as a putative CUX1‐binding site (PCBS), which is located ~4.8 kb upstream of the transcription start site of *SATB2* (Figure 3h). To demonstrate that this PCBS is the binding site for CUX1, we first performed a DNA pulldown Western blot analysis. As shown in Figure 3i, a strong binding of CUX1 to the PCBS was observed; however, very little binding of CUX1 to the PCBS was detected with the CUX1 core binding motif mutated to ATCCAT. Next, we performed a luciferase reporter assay by cloning the PCBS (ATCAAT) into the promoter vector pGL3 (Promega) under the condition that CUX1 was downregulated. As it can seen in Figure 3j, a significant reduction in luciferase reporter activity was detected in the CUX1 knockdown cells compared to the scrambled shRNA control. While these data demonstrate that CUX1 is a transcription factor for *SATB2*, it also indicates that, besides the PCBS, CUX1 might need other element(s) on the genomic sequence in order to function as a transcriptional suppressor in repressing *SATB2* expression. To further demonstrate that CUX1 binding to the PCBS, we also performed a ChIP assay using anti‐CUX1 antibody to pull down the DNA fragment that contains the PCBS. As the results shown in Figure 3k, a significant increase in the binding of CUX1 to the PCBS was detected in comparison with the control using an isotype antibody. Taken together, our data suggest that while SATB2 suppresses p16^INK4a^ expression by competing with CUX1 for its binding to the fSNP rs1537371 in human ECs, SATB2 itself is also transcriptionally inhibited by CUX1.

### 2.5 SATB2 regulates p16^INK4a^ ‐dependent cellular senescence by competing with CUX1 for binding to the fSNP rs1537371

**FIGURE 4:**
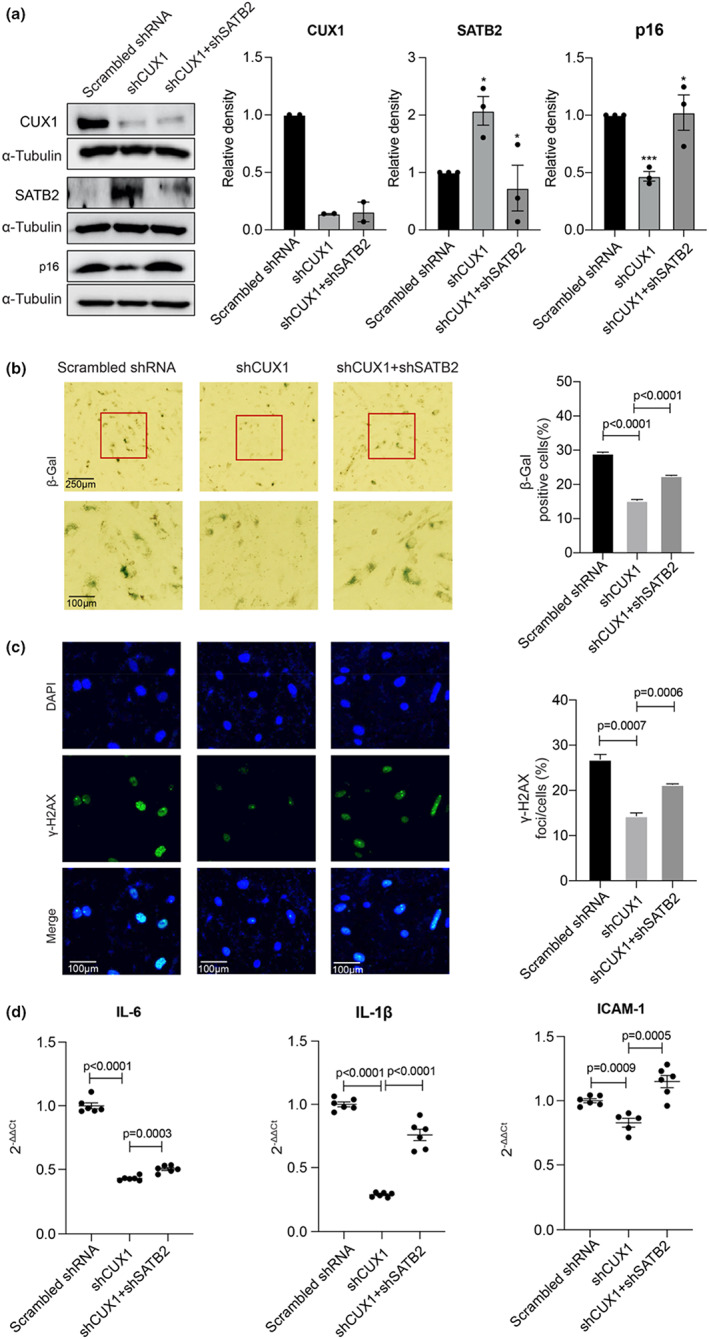
Functional analysis showing that SATB2 regulates cellular senescence in the *CUX1* shRNA knockdown human ECs by inactivating the *p16* ^*INK4a*^ expression. (a) Western blot analysis showing a significant upregulation of SATB2 and a downregulation of p16^INK4a^ in the *CUX1* shRNA knockdown ECs (left and middle lane) as well as the reversed expression of p16^INK4a^ by SATB2 shRNA knockdown in the *CUX1* shRNA knockdown human ECs (middle and right lane). Relative density of CUX1, SATB2 and p16^INK4a^ in the Western blots was shown. Data for CUX1 Western blot analysis represent two biologically independent experiments (*n* = 2) and data for SATB2 and p16^INK4a^ Western blot analysis represent three biologically independent experiments (*n* = 3). (b and c) SA‐β‐gal and γ‐H2AX staining showing a decreased cellular senescence in the *CUX1* shRNA knockdown ECs (left and middle panel) and a recovery of cellular senescence in the *CUX1* and *SATB2* double knockdown human ECs (middle and right panel). Quantitative plots for both β‐gal‐positive cells (%) following SA‐β‐gal staining and γ‐H2AX foci/cells (%) after γ‐H2AX staining are shown on the right side of the panel. Data for SA‐β‐gal and γ‐H2AX staining represent three biologically independent experiments (*n* = 3). (d) qPCR analysis showing a downregulation of SASP genes *IL‐6*, *IL‐1β* and *ICAM‐1* in the *CUX1* shRNA knockdown human ECs (left and middle lane) and a restoration of the expression of these SASP genes in the *CUX1* and *SATB2* double shRNA knockdown human ECs (middle and right lane). Data for qPCR analysis represent a combination of three biologically independent samples (*n* = 3), each performed in duplicate. sh: shRNA. *p‐value < 0.05; **p‐value < 0.01; ***p‐value < 0.001.

To demonstrate that SATB2 and CUX1 coordinately regulate cellular senescence by modulating p16^INK4a^ expression, we first downregulated CUX1 by shRNA in ECs (Figure 4a, left and middle lane, upper). As a consequence, an upregulation of SATB2 and a downregulation of p16^INK4a^ were observed in these cells (Figure 4a, left and middle lane, middle and lower). Correspondingly, decreased cellular senescence was detected in these ECs as evidenced by a decreased staining of both SA‐β‐gal and γ‐H2AX (Figure 4b,c, left and middle panel), as well as by a decreased expression of the SASP genes *IL‐6, IL‐1β* and *ICAM‐1* (Figure 4d, left and middle lane). While these data recapitulate our previous findings that CUX1 is an activator of cellular senescence via inducing p16^INK4a^ expression (Jiang et al., 2022), they also demonstrate that upregulation of SATB2 in CUX1 knockdown human ECs coordinately inactivates cellular senescence by downregulating p16^INK4a^ expression. Next, to demonstrate the competitive nature between SATB2 and CUX1 in regulating cellular senescence, we downregulated SATB2 by shRNA lentivirus in the CUX1 shRNA knockdown ECs (Figure 4a, middle and right lane, upper and middle). As predicted, an upregulation of p16^INK4a^ was detected in the CUX1 and SATB2 double knockdown ECs (Figure 4a, middle and right lane, lower). Consistent with the induction of the p16^INK4a^ expression, we observed a recovery of cellular senescence in these ECs to almost the same level as the scrambled control ECs as evidenced by an increase in both SA‐β‐gal and γ‐H2AX staining (Figure 4b,c, middle and right panel), as well as in the expression of the SASP genes *IL‐6, IL‐1β*, and *ICAM‐1* (Figure 4d, middle and right lane). This indicates that SATB2 competes with CUX1 to regulate p16^INK4a^‐dependent cellular senescence presumably via the fSNP rs1537371.

These data, together with the data presented above, demonstrate that SATB2 is a transcriptional target of CUX1 and inhibits p16^INK4a^‐dependent cellular senescence by competing with CUX1 for binding to the fSNP rs1537371 in human ECs. Therefore, decreased expression of SATB2 in the CUX1 upregulated cells results in a coordinative binding of SATB2 with CUX1 to the fSNP rs1537371 for fine‐tuning the expression of p16^INK4a^ and for cellular senescence.

### 2.6 IL‐1β induces a senescence‐like phenotype in human ECs by activating p16^INK4a^ expression via upregulating CUX1 and/or downregulating SATB2 in human ECs

**FIGURE 5:**
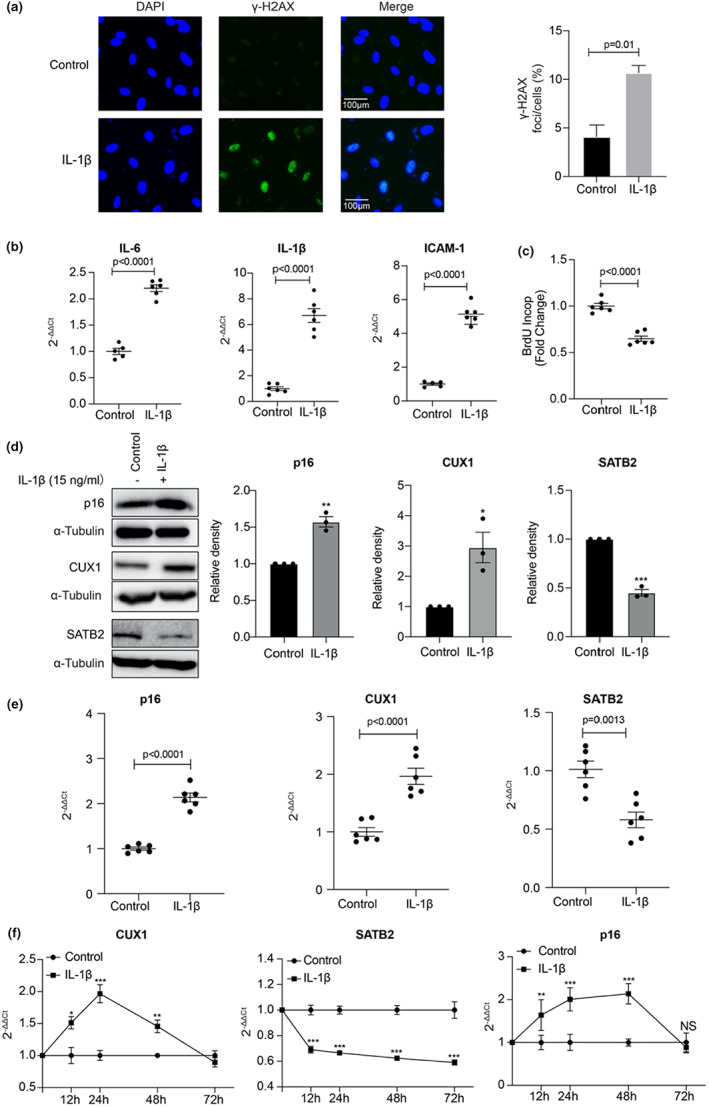
IL‐1β induces a senescence‐like phenotype in human ECs. (a) An increased γ‐H2AX staining in human ECs treated with 15 ng/ml IL‐1β for 24 h. quantitative plots for γ‐H2AX foci/cells (%) after γ‐H2AX staining are shown on the right side of the panel. Data for γ‐H2AX staining represent three biologically independent experiments (*n* = 3). (b) qPCR analysis demonstrating a significant increase in the expression of the SASP genes *IL‐6, IL‐1β*, and *ICAM‐1* in human ECs treated with IL‐1β. Data for qPCR analysis represent a combination of three biologically independent samples (*n* = 3), each performed in duplicate. (c) BrdU incorporation assay showing a defect in cell proliferation in human ECs treated with IL‐1β. Data for BrdU incorporation assay represent a combination of three biologically independent samples (*n* = 3), each performed in duplicate. (d) Western blot analysis showing an upregulation of p16^INK4a^ and CUX1 and a downregulation of SATB2 in the human ECs treated with 15 ng/ml IL‐1β for 24 h. Relative density of CUX1, SATB2 and p16^INK4a^ in the Western blot was shown. Data for the Western blot analysis represent three biologically independent experiments (*n* = 3). (e) qPCR analysis showing an upregulation of p16^INK4a^ and CUX1 and a downregulation of SATB2 in the human ECs treated with 15 ng/ml IL‐1β for 24 h. Data for qPCR analysis represent a combination of three biologically independent samples (*n* = 3), each performed in duplicate. (f) Time courses of *CUX1*, *SATB2* and *p16* ^*INK4a*^ expression in the human ECs treated with 15 ng/ml IL‐1β for 12, 24, 48 and 72 h, respectively, as shown by qPCR analysis. All the qPCR data were normalized to the untreated control. Data for qPCR analysis represent a combination of three biologically independent samples (*n* = 3), each performed in duplicate. *p‐value <0.05; **p‐value <0.01; ***p‐value <0.001

To determine whether IL‐1β plays a role in the induction of cellular senescence in human ECs as a causal condition of atherosclerosis, human ECs treated with IL‐1β at a concentration of 15 ng/ml for 24 h were stained by both SA‐β‐gal and γ‐H2AX. Although we did not observe any obvious change in SA‐β‐gal staining at this condition, to our surprise, a dramatic induction of γ‐H2AX staining was detected (Figure 5a). Consistently, an increased expression of the SASP genes including *IL‐6, ICAM‐1* and *IL‐1β* itself (Figure 5b) and a defective cell proliferation (Figure 5c), as two more senescence markers, were detected in the IL‐1β‐treated human ECs by qPCR and BrdU incorporation, respectively. Together, these data suggest that IL‐1β is an inducer that can activate a senescence‐like phenotype in human ECs.

To understand how IL‐1β induces a senescence‐like phenotype in human ECs., we first checked the expression of p16^INK4a^, another marker for cellular senescence, in the IL‐1β‐treated human ECs under the same condition (15 ng/ml for 24 h). A significant induction of p16^INK4a^ was observed at both the protein and mRNA levels (Figure 5d,e). To test the possibility that IL‐1β‐induced expression of p16^INK4a^ is regulated via SATB2 and/or CUX1, we performed Western blot and qPCR on these two genes using the same IL‐1β‐treated human ECs. Remarkably, upregulation of CUX1 and downregulation of SATB2 were observed in the IL‐1β‐treated human ECs compared to the untreated control ECs (Figure 5d,e). These data suggest that IL‐1β may induce a senescence‐like phenotype by activating p16^INK4a^ expression via upregulating CUX1 and/or downregulating SATB2.

Currently, we do not know whether SATB2 is directly inhibited by IL‐1β or not. However, these data are consistent with our finding shown in Figure 3 that CUX1 is a transcriptional suppressor of SATB2. To fully understand how IL‐1β modulates the expression of CUX1, SATB2, and p16^INK4a^, we performed a time course analysis on the expression of CUX1, SATB2, and p16^INK4a^ induced by IL‐1β. Our qPCR results, as shown in Figure 5f, indicate that both *CUX1* and *p16* ^*INK4a*^ were induced by 15 ng/ml IL‐1β at 12 h, with a peak at 24 h for CUX1 and a peak at 48 h for p16^INK4a^. At 72 h, the expression of both *CUX1* and *p16* ^*INK4a*^ were restored to the untreated level. On the contrary, our results also show that *SATB2* was suppressed at 12 h of IL‐1β treatment and stayed at the same level until the 72 h time point.

To demonstrate that the CUX1/SATB2/ p16^INK4a^ pathway is responsible for IL‐1β‐induced senescence‐like phenotype, we also performed functional complementation assays by either downregulating CUX1 or over‐expressing SATB2 in the IL‐1β treated human ECs (Figure S7a,b). As expected, IL‐1β treatment induced the expression of p16^INK4a^ and both downregulation of CUX1 and upregulation of SATB2 in the IL‐1β‐treated human ECs reduced the expression of p16^INK4a^ to a level that is equivalent to that in the control cells (Figure S7a,b, middle and right lane). Consistent with the p16^INK4a^ expression profile, we observed a restoration of cellular senescence as evidenced by a reduced γ‐H2AX staining and a decreased SASP gene expression (Figure S7c,d, second from the left, second from the right and right panel). All these data, together with the data presented in Figure 5, demonstrate that IL‐1β can induce a senescence‐like phenotype at least in human ECs via activating p16^INK4a^ expression by either upregulating CUX1 and/or downregulating SATB2.

## 3 DISCUSSION

Previously, we demonstrated that CUX1 activates p16^INK4a^‐dependent cellular senescence in human ECs via binding to the atherosclerosis‐associated fSNP rs1537371, with the risk allele A binding more CUX1 than the non‐risk allele C (Jiang et al., 2022), which reveals a mechanism underlying the contribution of the atherosclerosis‐associated fSNP rs1537371 to the susceptibility of this disease. In this report, we demonstrate the same specific binding of SATB2 to the fSNP rs1537371, with also the risk allele A binding more SATB2 than the non‐risk allele C. However, unlike CUX1, SATB2 is a suppressor of p16^INK4a^ and binding of SATB2 to rs1537371 results in downregulation of p16^INK4a^ expression and inhibition of cellular senescence. Therefore, SATB2 can compete with CUX1 in regulating p16^INK4a^ expression and endothelial senescence. Interestingly, we also find that CUX1 is a transcriptional suppressor of SATB2, and its expression can decrease the binding of SATB2 to rs1537371 by reducing the expression of SATB2. Thus, SATB2, coordinated with CUX1, fine‐tunes the expression of p16^INK4a^, which ensures a precise and tight regulation of cellular senescence.

Both experimental and clinical evidence support that IL‐1β is an important contributor to atherosclerosis and its complications (Libby, 2017). However, the underlying mechanism is not yet well‐understood. In this report, we find that IL‐1β can induce a p16^INK4a^‐dependent senescence‐like phenotype in human ECs as evidenced by increased γ‐H2AX staining, upregulation of both p16^INK4a^ and SASP gene expression, as well as arrested cell cycle. We further demonstrate that IL‐1β induces a senescence‐like phenotype in human ECs also via activation of CUX1 and/or suppression of SATB2. However, currently, we do not know the signal transduction pathway that IL‐1β activates CUX1 and/or suppreses SATB2. We also do not know how IL‐1β is activated at first place in the development of atherosclerosis.

Previously, we also demonstrated that induction of cellular senescence by telomeric shortening, DNA damage induced by bleomycin, and oxidative stress as mimiced by H_2_O_2_ at least in human ECs uses the same CUX1/p16^INK4a^ pathway as IL‐1β does, resulting in upregulation of SASP genes including IL‐1β itself (Jiang et al., 2022). Cellular senescence induced by the abovementioned stresses is now recognized as primary senescence in contrast to secondary senescence that is induced by primary senescent cells either via cell‐to‐cell direct contact (juxtacrine), or through the secretion of SASP (paracrine) (Admasu et al., 2021). While it still remains to be determined if secreted IL‐1β during primary senescence can paracrinely activate secondary senescence both in vitro and in vivo, our findings suggest that secondary senescecne induced by IL‐1β in human ECs and/or VSMCs might be one of the machnisms that contribute to the accumulation of senescent cells, which could lead to atherosclerosis and its complications.

**FIGURE 6:**
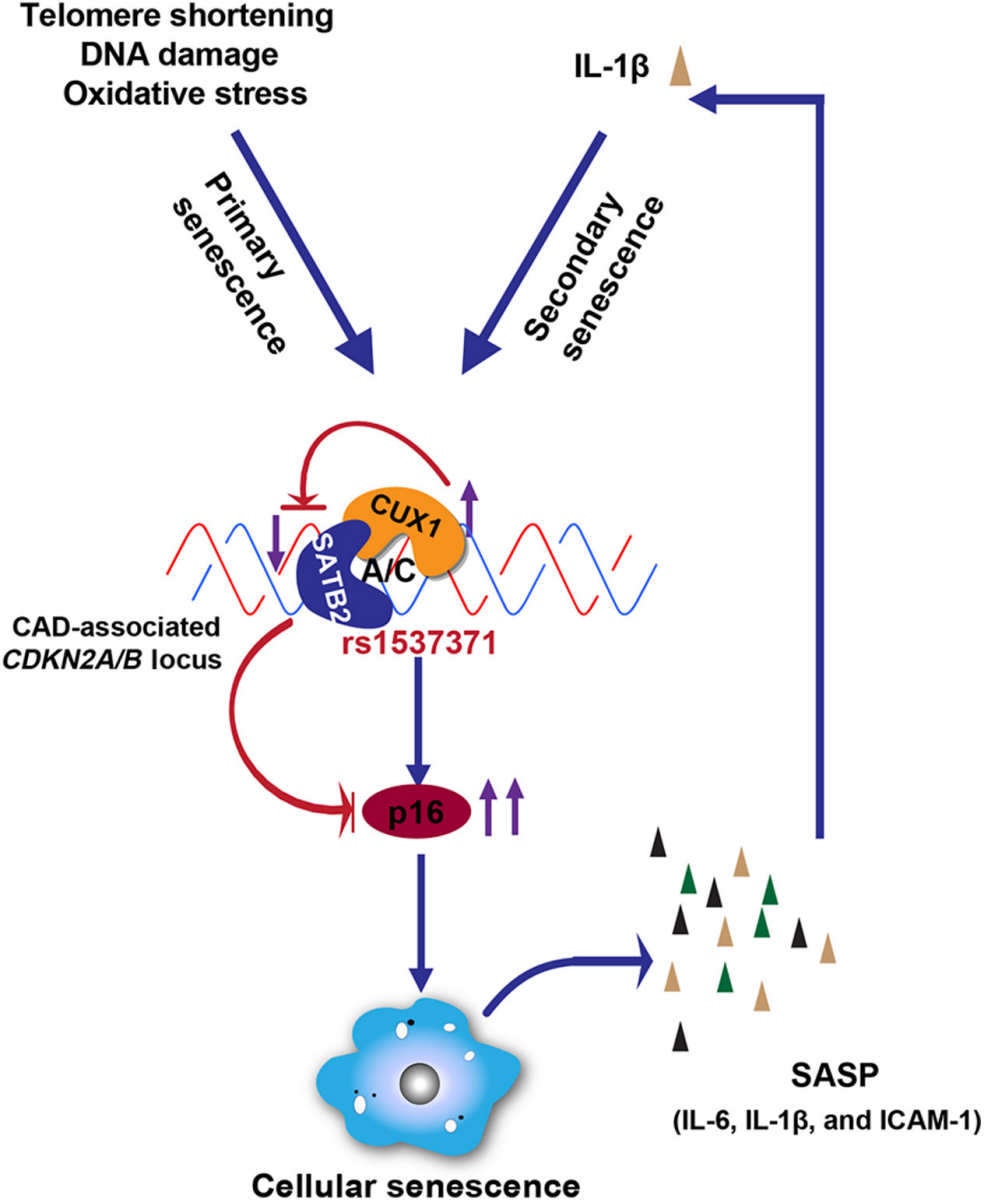
Model highlighting the CUX1/SATB2/ p16^INK4a^ signal transduction pathway leading to cellular senescence in human ECs via the atherosclerosis‐associated fSNP rs1537371 on the CAD‐associated *CDKN2A/B* locus. In human ECs, both stress‐induced primary sensencece and IL‐1β‐induced secondary senescence are activated via upregulation of CUX1 expression. However, it is not known how CUX1 is upregulated. Upregulation of CUX1, which suppresses SATB2 expression, enhances the binding of CUX1 to the atherosclerosis‐associated fSNP rs1537371(A/C) by reducing the ability of SATB2 to compete for the binding. Therefore, coordinated binding of CUX1 and SATB2 to the fSNP rs1537371 fine‐tunes p16^INK4a^ expression, which guarantees a precise and tight regulation of p16^INK4a^‐dependent endothelial senescence.

Collectively, based on our current work together with our previous findings (Jiang et al., 2022), we believe that both stresses‐induced primary senescence and IL‐1β‐induced secondary senescence at least in human ECs could be through activation of CUX1 expression as shown in the model described in Figure 6. This model also reveals that CUX1 is a transcriptional suppressor of SATB2 and upregulation of CUX1 can result in downregulation of SATB2. Therefore, together, CUX1 and SATB2 fine‐tune the expression of p16^INK4a^ via their competitive, but also coordinated binding to the atherosclerosis‐associated fSNP rs1537371, which precisely and tightly regulates cellular senescence in human ECs. Considering that endothelial senescence is the initial step of developing atherosclerosis (Davignon & Ganz, 2004) and IL‐1β is an important mediator and biomarker of atherosclerosis and a SASP gene itself, we believe that understanding the mechanism and the signal transduction pathway of endothelial senescence as we present in this model will give us new insights into the pathogenesis of atherosclerosis. Of note, we present this model based on our post‐GWAS functional analysis on the atherosclerosis‐associated fSNP rs1537371 (Hannou et al., 2015). However, as we revealed in Wu et al. (2021) (Wu et al., 2021) and Jiang and Sun et al. (2022) (Jiang et al., 2022), there are multiple other atherosclerosis‐associated fSNPs on the *CDKN2A/B* locus and hundreds of *cis*‐regulatory elements (*cis*‐REs) in the disease‐associated *CDKN2A/B* region. These *cis*‐REs could all regulate cellular senescence via modulating p16^INK4a^ expression. What are the regulatory proteins that bind to these *cis*‐REs and what are the signaling pathways that activate these regulatory elements? Do these *cis*‐REs coordinate with each other regulating p16^INK4a^‐dependent cellular senescence? Once these questions and many other questions are answered, we will have a better idea about how disease‐associated endothelial senescence is regulated, which, we believe, will provide us a better strategy to develop therapeutics for these diseases.

## 4 MATERIALS AND METHODS

### 4.1 Cell culture and reagents

Primary human arterial ECs (Cat#: CC‐2535) were purchased from Lonza. Cells were cultured at 37°C in 5% CO_2_ in basal medium EGM‐2 supplemented with 10% fetal bovine serum. All cells are free of mycoplasma.

### 4.2 Primers and antibodies

All primers used in this study were purchased from IDT and are listed in Table S1. All antibodies used are listed in Table S2 with the corresponding supplier information.

### 4.3 Isolation of atherosclerotic plaques

Atherosclerotic plaques were obtained from patients undergoing carotid endarterectomy at the Department of Surgery at UPMC as part of their standard‐of‐care. The use of deidentified human materials was approved by the University of Pittsburgh under a “No Human Subject Involvement” designation with the IRB number: PRO18060512.

### 4.4 ChIP assay

ChIP was performed as described previously (Noss et al., 2015). Briefly, scrambled shRNA control human ECs and the *SATB2* shRNA knockdown human ECs were cross‐linked with 1% formaldehyde for 10 min. Sonication was carried out at 30% amplitude with 20 s on and 50 s off for 5 min. Ten micrograms anti‐SATB2 antibody coupled to Dynabeads™ Protein A/G (Thermo Fisher Scientific, Cat#:10001D and 10003D) was incubated with sonicated nuclei at 4°C overnight. DNA was pulled down and purified using PCR purification kit (Macherwy‐nagel Cat#: 740609.5) after reversal of the crosslink. The purified DNA was then used for real‐time PCR analysis of the sequences around the fSNP rs1537371. Rabbit IgG was used as an isotype control. The data represent the combination of three independent samples (*n* = 3).

### 4.5 Luciferase reporter assay

Luciferase reporter assay was performed in 293 T cells using pGL3 luciferase reporter vector (Promega, cat#: E1761). Insert target sequences are listed in Table S1. Luciferase reporter construct DNA was transfected into 293 T cells by FuGENE HD transfection reagent (Promega, Cat#: E2311) together with the control vector. Luciferase activity was measured by the Dual‐Glo® Luciferase Reporter Assay System (Promega, Cat#: E2920). All experiments were performed according to the manufacturer's protocol. The data represent six independent biological replicates (*n* = 6).

### 4.6 AIDP‐Wb analysis

AIDP‐Wb was performed as previously described (Zhao et al., 2020). In brief, a 31‐bp biotinylated SNP sequence centered with either the risk or non‐risk allele was generated by annealing two biotinylated primers (IDT). Approximately 1 μg DNA was then attached to 40 μl of Dynabead™M‐280 Streptavidin. DNA‐beads were mixed with ~100 μg nuclear extract isolated from ECs at RT for 1 h with rotation. Nuclear extracts were isolated using NE‐PER Nuclear and Cytoplasmic Extraction Reagents (Thermo Scientific, Cat#: 78835). After washing off the unbound proteins, the DNA bound proteins were eluted by sample buffer and resolved on an SDS‐PAGE gel for Western blot analysis using an antibody directed against SATB2. For an internal control, the same blot was probed using an antibody directed against PARP‐1. The data represent three independent biological replicates (*n* = 3).

### 4.7 qPCR analysis

Total RNA was isolated using the RNeasy Mini kit (Qiagen). cDNA was synthesized using SuperScript® III Reverse Transcriptase (Invitrogen) after the RNA samples were treated with DNase I (Invitrogen). All the procedures were performed following the manufacturer's protocols. qPCR was performed with the StepOne real‐time PCR system according to the protocol for the Power SYBR Green PCR Master Mix (Applied Biosystems) and for TaqMan Universal PCR Master Mix (Applied Biosystems). The following probe/primer mixes for TaqMan PCR were purchased from Applied Biosystems: p14 Hs99999189_m1; p15 Hs00793225_m1; p16 Hs02902543_mH; *ANRIL* Hs04259472_m1 and GAPDH internal control (Hs02786624_g1). Other Primers used are listed in Table S1. The data represent the combination of three independent samples (*n* = 3).

### 4.8 Western blot analysis

Whole cell lysates prepared using RIPA buffer (Sigma, Cat#: 89900) were used for Western blot analysis. Proteins were resolved on SDS‐PAGE gels, transferred to PVDF membranes and then detected using gene‐specific antibodies. All antibodies were purchased and used as listed in Table S2. For a loading control, α‐Tubulin was used. The data represent three independent biological replicates (*n* = 3).

### 4.9 Senescence‐associated β‐galactosidase staining

SA‐β‐Gal Staining Kit (Cell Signaling, Cat#: 9860 S Danvers, MA, USA) was used to stain senescent ECs. Visualization was done using an RVL‐100‐G microscope (Echo Laboratories, San Diego, CA, USA). Images were analyzed using ImageJ software (version 1.52 K, NIH). The data represent three independent biological replicates (*n* = 3).

### 4.10 γ‐H2AX staining

Cells were plated on glass coverslips and fixed in 4% paraformaldehyde. For γ‐H2AX staining, cell membrane was solubilized in PBS containing 5% FBS and 0.5% Triton X‐100. Cells were first incubated with γ‐H2AX antibodies in the solubilizing buffer for 1 hr and immunofluorescence was detected with Alexa Fluor 488‐conjugated secondary antibody. Cells were counterstained with 4′, 6‐diamidino‐2‐phenylindole (DAPI) (Sigma, Cat#: D9542). Visualization was carried out using an RVL‐100‐G microscope (Echo Laboratories, San Diego, CA, USA). Images were analyzed using ImageJ software (version 1.52 K, NIH). The data represent three independent biological replicates (*n* = 3).

### 4.11 RNAi knockdown

For siRNA transient knockdown in human ECs, siRNAs for human *SATB2*, *CUX1* and *p16* ^*INK4a*^ were purchased from Horizon Discovery and knockdown was performed according to the manufacturer's protocol. For *SATB2* and *CUX1* shRNA knockdown in human ECs, lentiviruses were generated using the pLKO.1‐puro vector (Addgene, Plasmid #8453). Assays for cellular senescence including SA‐β‐Gal and γ‐H2AX staining as well as SASP gene expression were performed 48 h after siRNA transfection or shRNA lentiviral infection. The targeted sequences are listed in Table S1.

### 4.12 Overexpression of SATB2, CUX1 and p16^INK4a^

For overexpression of human SATB2 and CUX1, human SATB2 and CUX1 cDNAs were cloned into the pLVX‐puro vector (Clontech, Cat#: 632164) and confirmed by sequencing. p16^INK4a^ was overexpressed using lentiviral expression vector pLenti CMV p16 Neo (Addgene, Cat#: W111‐1). Lentiviruses were generated by transfecting 293 T cells and used to infect human ECs. Assays for cellular senescence including SA‐β‐Gal and γ‐H2AX staining as well as SASP gene expression were performed 48 h after viral infection.

### 4.13 Immunocytochemical staining for p16^INK4a^ and SATB2

Human atherosclerotic plaques were obtained from patients undergoing carotid endarterectomy. Part of the carotid artery that shows calcified hard tissue was used as a plaque zone and part that is far from the calcified zone was used as normal‐appearing zone. Both plaque and normal‐appearing zones were separated and fixed with 4% buffered formalin for 2 h and stored in 30% sucrose solution containing 0.05% sodium azide overnight. Sections were made of 10 micrometers thickness, permeabilized with 0.1% triton X‐100 for 4 h, and blocked overnight in PBS containing 2% BSA. Sections were further incubated for 24 h with primary antibodies against p16^INK4a^ (Invitrogen, cat#: MA5‐17142; 1:500 dilution) and SATB2 (Novus Biologicals Cat#: NBP1‐03328; 1:1000 dilution). After washing with PBS, sections were incubated for 1 h at room temperature with fluorochrome conjugated secondary antibodies (Alexa fluor‐488 goat anti‐mouse and Alexa fluor‐647 goat anti‐ rabbit). Tissue sections were stained and mounted with VECTASHIELD DAPI. Images were taken using confocal laser microscopy and analyzed using image J. The data represent two independent experiments with eight plaque zones (*n* = 8) and eight normal‐appearing zones (*n* = 8).

### 4.14 Statistical analysis

For normally distributed data, all data were represented as standard error mean (SEM). *p*‐values were calculated using Student's *t*‐test with two tails. The non‐normally distributed data related to the quantification of SATB2 and p16^INK4a^ immunocytochemical staining in Figure 3 were represented as Mean ± SEM interquartile range, and *p*‐values were calculated with the non‐parametric Mann–Whitney test for pairwise comparisons.

## AUTHOR CONTRIBUTIONS

Gang Li designed the study, analyzed the data, and drafted and revised the manuscript; Ting Wu, Yuwei Wu, and Danli Jiang performed all the experiments and participated in drafting the manuscript; Wei Sun and Meijuan Zou assisted with the experiments; Sathish Babu Vasamsetti and Partha Dutta performed immunocytochemical staining. Steven A. Leers collected human samples. Wu Di performed sequencing data and statistical data analysis.

## FUNDING INFORMATION

This work was supported partly by grants from NIH NIA R01AG056279 (GL) and R01AG065229 (GL).

## CONFLICT OF INTEREST

The authors declare no competing financial interests.

## Supporting information


SupinfoS1

## Data Availability

All data and reagents are available upon reasonable request.
