# Supplementary material for: SATB2, coordinated with CUX1, regulates IL‐1β‐induced senescence‐like phenotype in endothelial cells by fine‐tuning the atherosclerosis‐associated p16INK4a expression
Source: Aging Cell. 2023 Jan 12;22(2):e13765. doi: 10.1111/acel.13765 (PMC9924951; doi:10.1111/acel.13765)
Supplement: Supplementary file 1 — SupinfoS1 [file ACEL-22-e13765-s001.docx]

**Supplementary Figures**

**Supplementary Figure 1. Characterization of the expression of *p14^ARF^, p15^INK4b^, p16^INK4a^* and ANRIL by SATB2 siRNA knockdown. A.** qPCR analysis showing that knockdown of *SATB2* by siRNA results in a downregulation of *p14****^ARF^*** and ANRIL*,* and an upregulation of *p15^INK4b^* and *p16^INK4a^* in primary human EC. Data for qPCR analysis represent a combination of three biologically independent samples (n=3), each performed in triplicate. **B**. Cell proliferation assay using cell number counting showing significantly decreased cell proliferation in the *SATB2* knockdown human ECs. **C**. Cell cycle analysis showing decreased cell number in S/G_2_/M phase in SATB2 knockdown human ECs (n=3). si: siRNA knockdown and sh: shRNA.


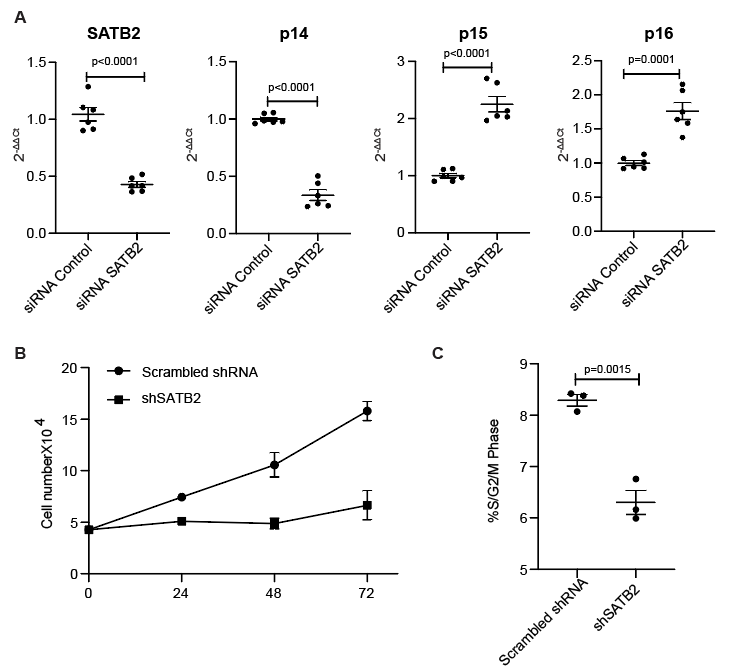


**Supplementary Figure 2. SA-β-gal staining in passage 8 ECs five days after shRNA SATB2 lentiviral infection.** sh: shRNA.


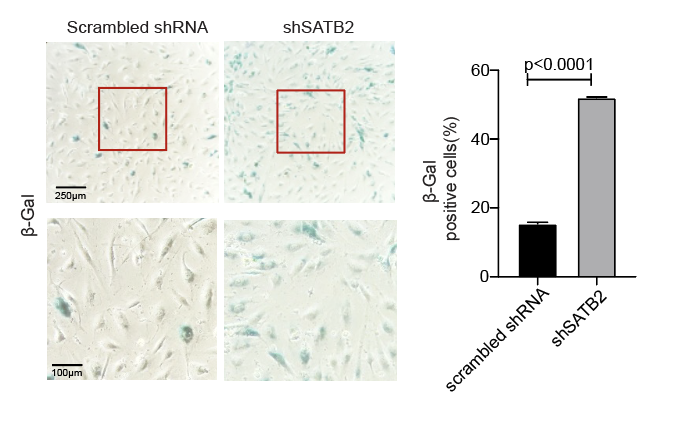


**Supplementary figure 3. Functional analysis demonstrating that overexpression of SATB2 inhibits cellular senescence by inactivating the p16^INK4a^ expression**. **A**. Western blot analysis showing a significant downregulation of p16^INK4a^ expression in the SATB2-overexpressed human ECs (**left** and **middle lane**), as well as ectopic overexpression of p16^INK4a^ in the SATB2 overexpressed human ECs (**middle** and **right lane**). Relative density of SATB2 and p16^INK4a^ in the Western blots was shown. Data for the Western blot analysis represent two biologically independent experiments (n=2)**. B.** SA-β-gal and **C.** γ-H2AX staining showing a decreased cellular senescence in the SATB2-overexpressed human ECs (**left** and **middle panel**) and a recovery of cellular senescence in the SATB2 and p16^INK4a^ double-overexpressed human ECs (**middle** and **right panel**) in the passage 10 human ECs. DAPI (blue) was applied to stain nuclei. Quantitative plots for both β-gal-positive cells (%) following SA-β-gal staining and γ-H2AX foci/cells (%) after γ-H2AX staining are shown on the right side of the panel. Data for SA-β-gal and γ-H2AX staining represent three biologically independent experiments (n=3). **D**. qPCR analysis showing the significant downregulation of SASP genes *IL-6*, *IL-1β* and *ICAM-1* in the SATB2-overexpressed human ECs (**left** and **middle lane**) and a restoration of the expression of these SASP genes in the SATB2 and p16^INK4a^ double-overexpressed human ECs (**middle** and **right lane**). Data for qPCR analysis represent a combination of three biologically independent samples (n=3), each performed in duplicate**.** pLVX: overexpression vector; pLVX SATB2 and pLVX p16: overexpression of SATB2 and p16.


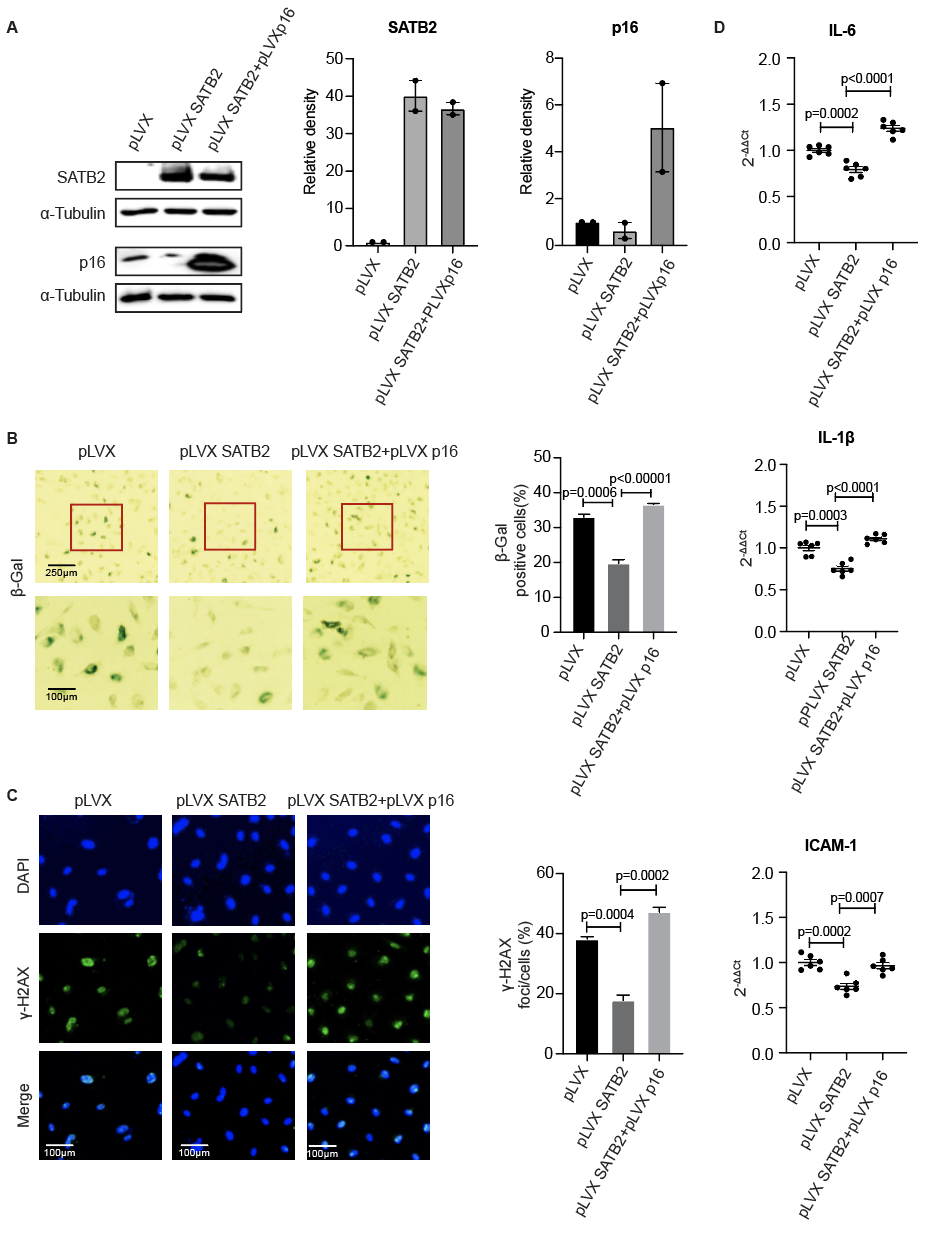


**Supplementary figure 4. Decreased SATB2 and increased p16^INK4a^ expression in the plaque zones from patients with carotid artery disease. A**. Immunocytochemical staining with antibodies specifically against SATB2 (purple) and p16^INK4a^ (green) in the plaque and normal-appearing zones from patients with carotid artery atherosclerosis. **B**. Quantitative analysis of the immunocytochemical staining in **A** showing a statistical significance of the reduction of SATB2 (*p-*value < 0.0001) and induction of p16^INK4a^ (*p-*value = 0.0035) in the plaque zone versus the normal-appearing zones. The data were generated by staining eight plaque zones (n=8) and eight normal-appearing zones (n=8) in two independent experiments. *p-*value was calculated by the non-parametric Mann-Whitney test for pairwise comparisons. DAPI (blue) was applied to stain nuclei.

**
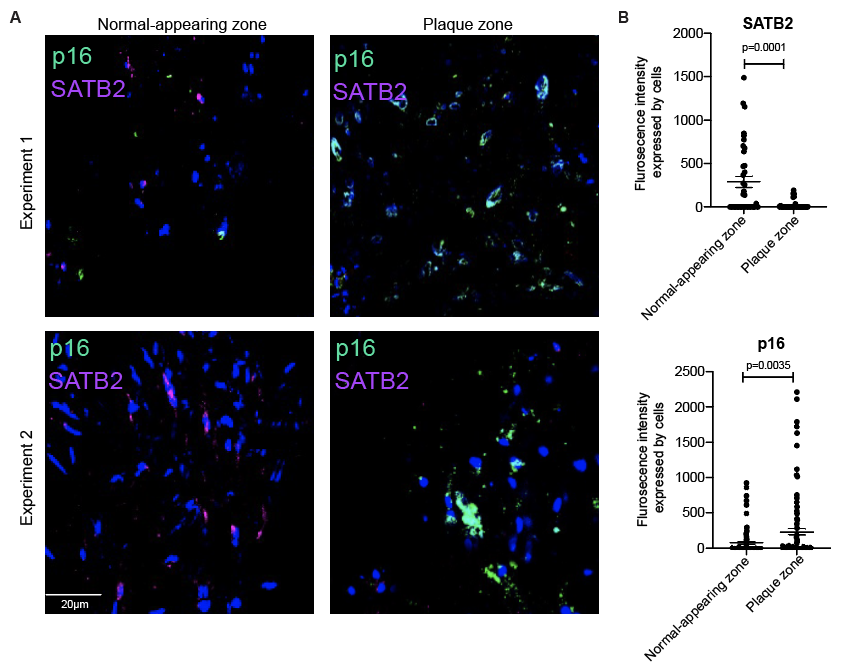
**

**Supplementary figure 5. Functional analysis showing that p15^INK4b^ is not the downstream factor of SATB2 in regulating cellular senescence in human ECs. A**. Western blot analysis and **B.** qPCR analysis showing a significant upregulation of p15^INK4b^ expression in the *SATB2* shRNA knockdown human ECs (**left** and **middle lane**) as well as knockdown of p15^INK4b^ in the *SATB2* shRNA knockdown human ECs (**middle** and **right lane**). Data for the Western blot analysis represent three biologically independent experiments (n=2). Data for qPCR analysis represent a combination of three biologically independent samples (n=3), each performed in duplicate**. C.** SA-β-gal and **D.** γ-H2AX staining showing an increased cellular senescence in the *SATB2* shRNA knockdown human ECs (**left** and **middle panel**) and no significant recovery of cellular senescence in the *SATB2* and *p15^INK4b^* double knockdown human ECs (**middle** and **right panel**). Quantitative plots for both β-gal-positive cells (%) following SA-β-gal staining and γ-H2AX foci/cells (%) after γ-H2AX staining are shown on the right side of the panel. Data for SA-β-gal and γ-H2AX staining represent three biologically independent experiments (n=3). **E**. qPCR analysis showing a significant upregulation of the SASP genes *IL-6*, *IL-1β* and *ICAM-1* in the *SATB2* shRNA knockdown human ECs (**left** and **middle lane**). However, there is no obvious change in the expression of these SASP genes in the *SATB2* and *p15^INK4b^* double knockdown human ECs (**middle** and **right lane**). Data for qPCR analysis represent a combination of three biologically independent samples (n=3), each performed in duplicate**.** sh: shRNA knockdown.


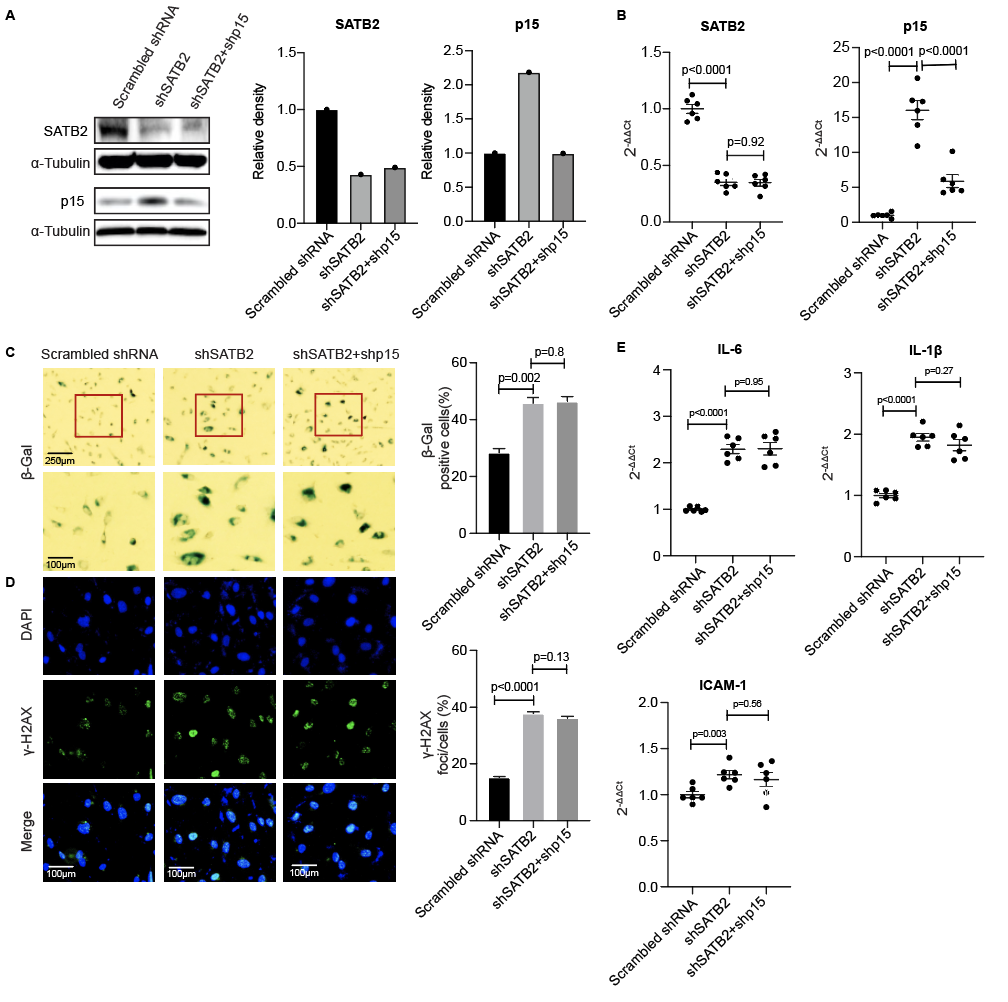


**Supplementary Figure 6. Functional analysis showing that ANRIL is not a regulator of cellular senescence in human ECs. A**. qPCR showing a significant downregulation of ANRIL expression by siRNA in human ECs. Data for qPCR analysis represent a combination of three biologically independent samples (n=3), each performed in duplicate**. B.** SA-β-gal and **C.** γ-H2AX staining showing no obvious change of cellular senescence in the ANRIL siRNA knockdown human ECs. Quantitative plots for both β-gal-positive cells (%) following SA-β-gal staining and γ-H2AX foci/cells (%) after γ-H2AX staining are shown on the right side of the panel. Data for SA-β-gal and γ-H2AX staining represent three biologically independent experiments (n=3). si: siRNA knockdown.


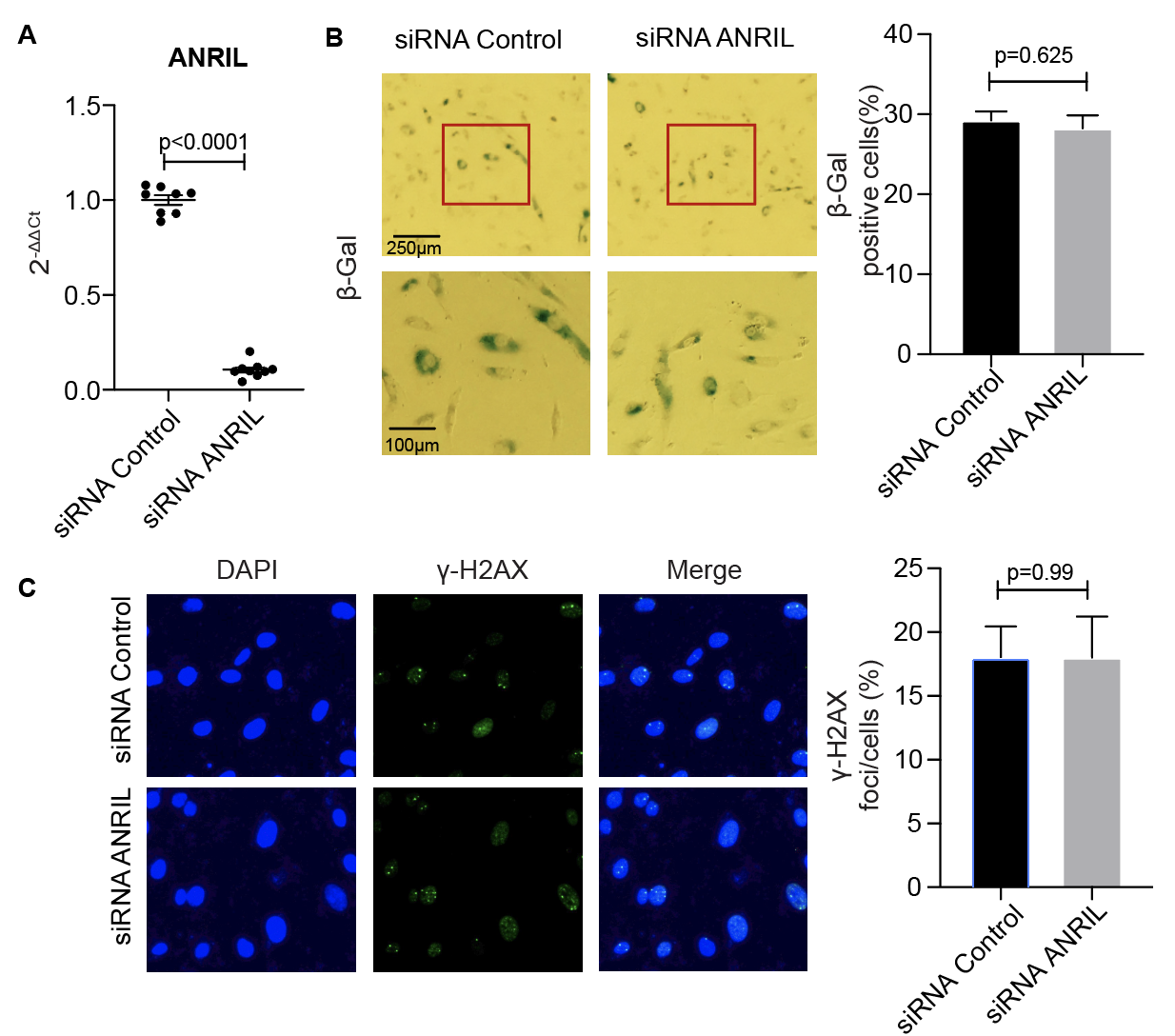


**Supplementary Figure 7. Functional complementational analysis confirms that CUX1 and SATB2 are the downstream mediators of IL-1β-induced senescence-like phenotype**. **A**. and **B**. Western blot analysis showing that IL-1β induces upregulation of p16^INK4a^ by activating CUX1 and/or suppressing SATB2. Upregulation of p16^INK4a^ can be inhibited by either CUX1 shRNA knockdown or SATB2 overexpression. The relative density of CUX1, SATB2 and p16^INK4a^ in the Western blot was shown. Data for Western blot analysis represent two biologically independent experiments (n=2). **C**. An increased γ-H2AX staining induced by IL-1β in human ECs and IL-1β-induced γ-H2AX staining can be restored by either downregulation of CUX1 or overexpression of SATB2 in human ECs. Data for γ-H2AX staining represent three biologically independent experiments (n=3). Quantitative plots for γ-H2AX foci/cells (%) after γ-H2AX staining are shown on the right side of the panel. **D**. An increased expression of the SASP genes *IL-6*, *IL-1β* and *ICAM-1* in the human ECs treated with 15 ng/ml IL-1β for 24 h indicating a senescence-like phenotype induced by IL-1β in human ECs and IL-1β-induced senescence-like phenotype can be restored by either downregulation of CUX1 or overexpression of SATB2 in human ECs. Data for qPCR analysis represent a combination of three biologically independent samples (n=3), each performed in duplicate. pLVX SATB2: overexpression of SATB2 and sh: shRNA.


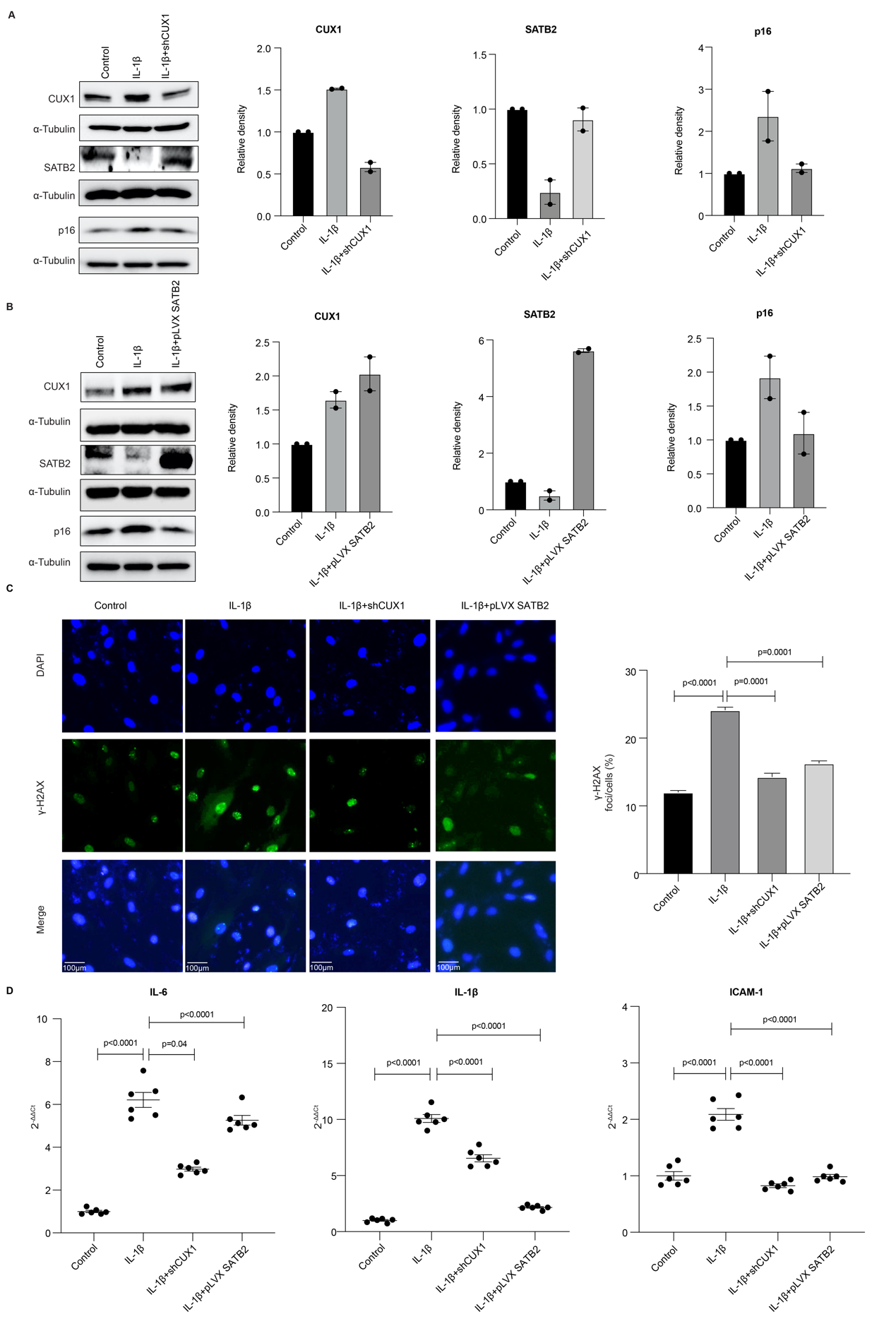


**Supplementary Table 1. Primers used in this paper**

| **Usage** | **Primer name** | **Sequence** |
| --- | --- | --- |
| QPCR | IL6-F | GCAGAAAACAACCTGAACCTT |
|  | IL6-R | ACCTCAAACTCCAAAAGACCA |
|  | IL1b-F | ACAGATGAAGTGCTCCTTCCA |
|  | IL1b-R | GTCGGAGATTCGTAGCTGGAT |
|  | ICAM1-F | AGCGGCTGACGTGTGCAGTAAT |
|  | ICAM1-R | TCTGAGACCTCTGGCTTCGTCA |
|  | GAPDH-F | CGACCACTTTGTCAAGCTCA |
|  | GAPDH-R | AGGGGTCTACATGGCAACTG |
|  | SATB2-F | ATGTGAGCATGGTCTCCTCG |
|  | SATB2-R | GCGCCGTCCACCTTAATAG |
|  | CUX1-F | CCATGGAGTTTGCACCGT |
|  | CUX1-R | CAGCGAGCGGTTCTTCTC |
| shRNA knockdown | SATB2 shRNA | GCCATGCAGAATTTCCTCAAT |
|  | CUX1 shRNA | GCACGATATTGAAACAGAGAA |
| DNA pulldown Western blot | rs1537371-A | GGCATGTTATAATTTAATTGGCAGCATTATT |
|  | rs1537371-C | GGCATGTTATAATTTAATTGGCAGCATTATT |
|  | PCBS(ATCAAT) | AATAATTTAGAATCAATAGTTCCCTTCAAA |
|  | Control(ATCCAT) | AATAATTTAGAATCCATAGTTCCCTTCAAA |
| Luciferase reporter | rs1537371-A | GGCATGTTATAATTTAATTGGCAGCATTATT |
|  | PCBS(ATCAAT) | AATAATTTAGAATCAATAGTTCCCTTCAAA |
|  | Control(ATCCAT) | AATAATTTAGAATCCATAGTTCCCTTCAAA |
| ChIP | ChIP-F | GGGGAGCTGGGTTTGATAGC |
|  | ChIP-R | TGGGGGCCCTTAAGAGACAA |

**Supplementary Table 2. Antibodies used in this paper**

| **Antibody** | **Manufacturer** | **Cat#** | **Usage** |
| --- | --- | --- | --- |
| SATB2 | Novus Biologicals | NBP1-03328 | WB, ChIP |
| CUX1 | ABclonal | ABE217 | WB |
| CUX1 | Proteintech | 11733-1AP | ChIP |
| Rabbit IgG | Cell signaling | 2729S | ChIP |
| *p14^ARF^* | Invitrogen | MA5-14260 | WB |
| *p15^INK4b^* | Invitrogen | PA5-49749 | WB |
| *p16^INK4a^* | Proteintech | 10883-1-AP | WB |
| α-Tubulin | Sigma | T6074 | WB |
| PARP1 | Santa Cruz | Sc-7150 | WB |
| γ-H2AX | Santa Cruz | sc-517348 | IF |
| Alexa Fluor 488-conjugated antibodies | Invitrogen | A28175 | IF |
| anti-H3K4mel antibody | Active Motif | 61782 | ChIP |
